# Friedenheim Hospital

**Published:** 1902-05-31

**Authors:** 


### FRIEDENHEIM HOSPITAL.

The attendance at the annual general meeting was pronounced by an authority to be "very respectable indeed; in fact just right," for it was known to all friends and subscribers that the meeting of May 22nd was only preliminary to the great event of June 7th when H.R.H. Princess Christian was to open the new nurses' home.

The chair was taken by Mr. J. Herbert Tritton, who recalled the fact that the last year had opened with a considerable cloud over Friedenheim, but that at the present date that cloud had been to a great extent removed. Last year the new nurses' home was an object of their devout wishes—now it was an accomplished fact. Turning to the financial side of the question, the Chairman said that they began the year with a balance in hand of a substantial character, but he deprecated the idea that subscribers and friends should imagine that therefore funds were no longer needed; on the contrary, they were most urgently needed for the completion of the new building and the carrying on of the work. The Chairman drew attention to the nurses' home which had been erected at the back of the hospital and connected with it by a covered gallery. It was for their friends, he said, to place the top stone on the building so that it should be absolutely free from debt by the date of opening.

The hospital had during the past year lost the services as secretary of their valued friend Mr. Morton, and he had been replaced by Mr. P. J. Langdon. The meeting, said Mr. Tritton, would be completed on June 7th, when the new nurses' home was to be opened by Royalty.

Dr. Schofield, who was the next speaker, pointed out that a sum of about £3,000 was still needed to complete what was necessary at the nurses' home and also for certain alterations which the erection of the home had entailed in the main building. He hoped that their friends would bear this in mind and give them the happiness of knowing that the sum needed had been provided between that date and June 7th.

The Chairman, in terminating the proceedings, laid emphasis upon the fact that the thanks of all were due in the first place to Miss Davidson; they were there as her supporters, but the work was hers.

### Visit to the Nurses' Home.

After the conclusion of the meeting we were, as a special privilege, permitted to pay a brief visit to the new nurses' home. Connected by a covered way with the hospital, it is an entirely distinct, self-contained building, with a garden of its own, where the nurses will be able to have tea in the summer. Time was short, but a brief glimpse of the nurses' sitting-room, even in its present unfurnished condition, impressed us with a sense of cheerfulness and peace, and convinced us that it would prove a pleasant haven for the weary nurses after their sad and trying duties. Then we took a rapid survey of the bedrooms and bathrooms, till on the top storey of all we found the four bedrooms devoted to the night nurses, which are, through Miss Davidson's thoughtful care, separated by two glass partitions from the rest of the house, thus ensuring perfect quiet for those who have to sleep by day. "But surely,"

we asked, "all this will not be ready by June 7th?" "Certainly it will," was the prompt reply, "and furnished." It was so palpable that much yet remained to be done that we marvelled at so sanguine a forecast. Yet we could not help remarking that from the carpenter, engaged in some finishing touches, to the gardener who was making new paths in the nurses' garden, all, painters and varnishers alike, were working with a zest and heartiness that are not always characteristics of the British workman. Evidently they were inspired by the spirit of the home, and we felt that great as the task was, it would be duly accomplished by the appointed date. Electric light is fitted up in all the rooms and passages, and neither care nor money has been spared in order to render the nurses' home as complete in every detail as possible. And all this in the space of one brief year!

